# Co-creation of new knowledge: Good fortune or good management?

**DOI:** 10.1186/s40900-022-00394-2

**Published:** 2022-11-29

**Authors:** Tania Pearce, Myfanwy Maple, Kathy McKay, Anthony Shakeshaft, Sarah Wayland

**Affiliations:** 1 School of Health, University of New England, Armidale, NSW 2351 Australia; 2 Public Health, Policy and Systems, Institute of Population Health, University of Liverpool, Liverpool, UK; 3 Tavistock and Portman NHS Foundation Trust, London, UK; 4 National Drug and Alcohol Research Centre, University of New South Wales, Randwick Campus, 22-32 King Street, Randwick, NSW 2031 Australia

**Keywords:** Co-ideation, Co-design, Co-implementation, Co-evaluation, Multisectoral, Collaboration, third sector organisations, Patient and public involvement

## Abstract

**Background:**

Potential solutions to bridging the research practice gap include collaborative frameworks and models. Yet there is little evidence demonstrating their application in practice. In addressing this knowledge gap, this in-depth case study explored how the co-creation of new knowledge framework and its four collaborative processes (co-ideation, co-design, co-implementation, and co-evaluation) are utilised to support people who had attempted suicide through an Australian psychoeducational program known as Eclipse.

**Methods:**

Using a case study design and a thematic analysis methodology, multiple sources of qualitative data (collaborative group discussion, personal communications) were analysed inductively and deductively to examine the implementation of co-creation and explore the perspectives of researchers and stakeholders about co-creation and collaborative relationships.

**Results:**

Three broad themes were identified: (1) understanding the language and practice of co-creation, (2) perception of trust formation, and (3) the value of co-creation opportunities. Ultimately, implementing co-creation with or between researchers, industry and people with lived experience requires trust, reciprocity, good fortune, and good management. While implementing co-creation, the co-creation framework was revised to include additional elements identified as missing from the initially proposed framework.

**Conclusion:**

Co-creation of new knowledge poses many challenges to researchers and stakeholders, particularly regarding its “messiness” and non-linear approach to implementation and evaluation. However, as this case study demonstrates, it has the potential to become an alternative framework of best practice for public health interventions in third sector organisations, most notably as it eliminates the often-lengthy gap reported between research evidence and translation into practice. The research highlights the need for co-creation to further study its effectiveness in integrating research and service delivery to generate new knowledge. This may lead to a cultural and behavioural change in the service provider’s approach to research, offering better outcomes for providers, clients, policymakers, universities, and funders.

## Plain english summary

Organisations and researchers need to collaborate to produce new knowledge of health interventions. The literature identifies that there is a substantial evidence gap between producing knowledge and improving health outcomes. Here we reflect, via a case study methodology, on ways to co-create new knowledge by following a four-step collaborative process. The case study reviews the evaluation of an Australian-based psychoeducational program for people who attempt suicide by analysing multiple qualitative data sources to explore the perspectives of researchers and stakeholders. We discovered the need for a shared language of co-creation that focuses on experiences of collaboration while seeking out new value-creation opportunities and dismantling barriers. We learnt that implementing co-creation requires trust and good fortune within collaborative relationships alongside good management. Using the alternative collaboration framework of best practice for public health interventions in third sector organisations may eliminate gaps between research evidence and translation into practice, assisting health providers, clients, policymakers, universities, and funders.

## Background

Knowledge translation refers to ‘a dynamic and iterative process that includes the synthesis, dissemination, exchange, and ethically sound application of knowledge to improve health, provide more effective health services, and strengthen the health care system’ (1, p165). The production of knowledge and applying it to health interventions is sometimes perceived as a linear and unidirectional process. However, in reality, there is evidence of a substantial gap between the production of knowledge and improvement in health outcomes. A research-practice gap, or knowledge-action gap, describes the gap between what we know (research products) and what we do (actions) [2]. While researchers have employed various implementation and dissemination strategies to bridge this gap, it can be unclear how successful these attempts have been [3]. A linear, top-down approach to knowledge creation typically relies on researchers creating new evidence and using peer review as a primary method of sharing and communicating knowledge [4]. In practice, a complex interaction of systemic drivers often hampers the process of knowledge creation. In turn, this contributes to the research-practice gap by impeding or limiting the effectiveness of knowledge translation. Reported barriers to research translation include academics, practitioners and policymakers who operate from distinctive “communities of practice” with differing operational norms, values and priorities inhibiting research uptake [5, 6]. For third sector organisation (TSO) practitioners, a barrier is the lack of resources and time available to implement knowledge, a problem exacerbated by a lack of skills in research and evaluation [7]. The well-documented issue of a lack of collaborative practice between researchers and practitioners contributes to the research-practice gap [8, 9]. Various models, frameworks and approaches have been developed to overcome systemic barriers and improve the speed and efficiency of the research translation process [3].

One such approach is the co-creation of new knowledge (herein referred to as “co-creation”). Co-creation is regarded as an underutilised but complementary framework of research translation which holds great potential for reducing research waste and maximising research impact [10]. At the core of co-creation is the formation of collaborative partnerships between researchers (who have skills in evaluation and evidence translation), service providers (with skills in service delivery) and service users (with lived experience). In some contexts, this is known as PPI (Public and Patient Involvement); this paper is referred to as research and stakeholder (including industry and lived experience). Through these collaborative relationships, researchers and stakeholders work together across the research cycle to co-create knowledge that is both actionable and useable.

TSOs appear to be an ideal environment for applying frameworks such as co-creation. TSOs employ highly skilled service providers to implement health intervention programs and deliver services to end-users but lack an understanding of the technical aspects of the evaluation process, including data collection and analysis [7]. As a group, this makes them “research ready” to engage in collaborative relationships with researchers to solve complex problems through the mutual sharing of knowledge in research design and evaluation and the delivery of services [7, 11, 12]. Furthermore, by collaborating with TSO stakeholders, especially in suicide prevention, those with lived experience can engage researchers and contribute to improving services and program evaluations. Increasing collaborative engagement requires TSOs to participate in rigorous evaluations to demonstrate efficiency and effectiveness [13, 14]. Co-creating research offers TSOs a transparent evaluation process in which stakeholders and researchers communicate clearly at each stage of the four-step process. Apart from clear communication, the success of the co-creation process depends on good governance [15] and the establishment of an equitable and sustainable partnership founded on high levels of social capital and trust [15, 16].

Co-creation also has the potential to produce high-quality and cost-effective evaluations [10]. By integrating data collection with service delivery, co-creation can also enhance the research capacity and sustainability of TSOs [10]. Co-creation requires all parties, especially within the researcher-stakeholder collaboration, to work together through the program’s implementation from the conception stage through the program evaluation phase, undertaking four collaborative processes: “*(i) generating an idea (co-ideation); (ii) designing the program or policy and the research methods (co-design); (iii) implementing the program or policy according to the agreed research methods (co-implementation) and (iv) the collection, analysis and interpretation of data (co-evaluation)*” (10, p.11). The data collection process is an essential component of the co-creation framework, as it facilitates integrating research knowledge into delivering services to end users. For a full explanation of how co-creation is defined and constructed, refer to Pearce [10].

Whilst there exists an unspoken assumption of collaboration between researchers and TSOs, a recent systematic review of multisectoral collaborations in mental health and suicide suggests otherwise [17]. A review of 16 collaborative studies found no evidence that health-related TSOs engaged in co-creation or partnerships [17]. This paper presents a case study in which co-creation was operationalised with a TSO-based suicide prevention program to address the research-practice gap between researchers and practitioners. The study had three aims: (1) document and describe the events and critical factors influencing the implementation of co-creation, including the value of co-creation opportunities presented, (2) explore the perspectives of primary stakeholders, including researchers, to illustrate their understanding of the implementation of co-creation, and; (3) revisit the proposed model and make any adjustments.

### The case: eclipse program (lifeline mid coast)

Lifeline Mid Coast is a community-based TSO located in a semi-rural location on the North Coast of New South Wales (NSW), Australia, serving over 220,000 people [18]. This organisation specialises in the delivery of suicide prevention services and, in 2016, initiated discussions with researchers about implementing Eclipse, an 8 week psychoeducation group for people who had previously attempted suicide [19]. The Eclipse program was piloted in 2017 to reduce suicidality and increase resilience and help-seeking behaviours. The participant outcomes from the program are reported elsewhere. The Eclipse program was modelled on a similar program operated by a US mental health service, Didi Hirsch Mental Health Services [20], located in Los Angles, USA. Variations to the group curriculum for Australian and local context, adaptations to the evaluation tools, and Human Research Ethics Committee (HREC) approval were sought in 2017, then expanded to other sites outside Lifeline Mid Coast from 2018 onwards. Strong links with Didi Hirsch [20], the Australian researchers and service providers, have been maintained to share experiences of service delivery collaboratively. This case study presents the application of the co-creation framework, and its four collaborative processes, involved in the delivery and implementation of the Eclipse program.

## Methodology

### Case study design

According to Merriam [21], a case study is “an intensive, holistic description and analysis of a single instance, phenomenon or social unit”. Further to this, as suggested by Crowe [22], case studies are used to “generate an in-depth, multi-faceted understanding of a complex issue in its real-life context” In effect, they are focused on developing an in-depth understanding of “the whole” of a situation [22]. Given that this project called for an intensive investigation into applying a research translation framework to evaluating a TSO program over several years, a case study was deemed the most appropriate form of research design. Data sources for the case study include multiple documents and transcripts created throughout the project’s life and evidence from a Collaborative Group Discussion (CGD) held between researchers, TSO stakeholders and funders in March 2020. GCDs, as defined in this study, involve open discussions in which the researcher(s) play the dual role of both facilitator and participant. There are often challenges associated with researchers acting in a dual role. However, in this study, dual role tensions were minimised due to a previously established working relationship shared by the researchers, TSO stakeholders and funders. While the researcher guided the discussion, the discussion was not researcher-led. All participants (including the researchers) were encouraged to participate equally in the sharing of knowledge. The CGD represented an opportunity to collectively reflect on all experiences the researchers, the TSO stakeholders and the funders had encountered while working collaboratively over four years. Post-CGD discussions involved emails sent to stakeholders asking them to reflect on the co-creation framework and the previous collaborative group discussions.

### Key stakeholders identified for this case study

Across the project, the overall sample (n = 11) consisted of three different groups of participants: researchers (n = 3), TSO stakeholders, including peer workers with lived experience (n = 5) and funders (n = 3). Each group is described below, with their involvement highlighted.

1. Researchers: Three researchers were involved throughout the life of the project and participated in program planning and implementation discussions and activities. Two of the three researchers participated in the CGD, where they guided the discussion and shared their experiences and perspectives as co-participants in the research process.
2. TSO Stakeholders: This group consisted of two professionally trained facilitators in suicide prevention who were involved in the delivery of the Eclipse program, a TSO manager and two peer workers with lived experience. One of the peer workers was the program’s instigator, and one professionally trained facilitator (who retired during the project). A second professionally trained facilitator was recruited following the retirement of the first facilitator. Another TSO stakeholder participant included a TSO manager who had overseen the program’s establishment and development over time. A second peer worker joined the team halfway through the project.
3. Funders: The third group are the parent organisation’s employees funding the Eclipse evaluation (the Lifeline Research Foundation). The original foundation manager, his replacement, and the Foundation’s engagement manager participated.

### Human research ethics committee approval and consent to participate

Ethics approval was granted by the University of New England (HE16-219). Role titles are used as pseudonyms to protect the confidentiality of participants.

### Data collection and analysis

Various types of evidence, such as reports, reflective voice recordings, and CGD, were reviewed for relevant content to the co-creation activities (*the final sample consisted of 17 documents, we only examined documents with references to the four collaborative processes described earlier*)*.* Initially, ethics approval had been granted for the pilot testing of the Eclipse program from 2017 to 2018, permitting the collection of data, including reflective discussions between researchers, email correspondence between TSO stakeholders and researchers, and the production of program reports. Following this, ethical approval was extended to 2021, allowing for the data collection of CGD meetings and feedback from stakeholders associated with the TSO during that time. Before the CGD, participants received a copy of the information sheet and consent form. The CGD was held in March 2020 and co-facilitated by two researchers who also acted as co-participants. Using an interview guide and the co-creation framework, participants explored the impact of co-creation on the roles of TSO stakeholders and researchers and discussed the benefits and challenges of co-creation in program evaluation. The discussion was audio recorded and transcribed verbatim. Participants also received an invitation to share additional feedback about co-creation through follow-up email discussions. In addition to CGD and email feedback, transcribed reflections by the three researchers on the CGD outcomes were included in the analysis.

Data analysis was conducted through a hybrid deductive-inductive process, while a social constructionist perspective informed the interpretation of the data. We relied on the pre-existing co-creation framework [10] for the deductive analysis to identify co-creation-related activities. The data were also analysed using an inductive approach to uncover explicit meanings or responses (semantic) or conceptual themes that go beyond the mere description of data [23, 24]. Meanwhile, a thematic analysis aligns with the social constructionist paradigm, which perceives knowledge as being co-constructed between the researcher and the research participant (co-researchers), and accounts for the role of the researcher within the work. This study focuses on how participants, working within a co-creation framework, make sense of their experiences [23].

All text data relevant to the planning and implementation of the program (CGD transcripts, post-CGD emails, reports and transcribed reflective discussions) were uploaded into QSR NVivo 12. The data was deductively coded by TP using the co-creation framework [10] (see below), while inductive analysis was completed to capture any additional semantic and latent content. TP followed Braun and Clarke’s six-step thematic analysis process [24]. This process involved a recursive process of data familiarisation, deductive coding of data using the co-creation framework, thematic searching for additional semantic and latent responses, and reviewing and developing new themes as identified [24]. Preliminary coding results were discussed in-depth among all authors.

## Results

The case examined here allowed us to conduct an in-depth and multi-faceted exploration of the co-creation framework and its application to a TSO. In doing so, we analysed the CGD and post-CGD participant data to identify three primary themes. We also analysed case study material (as previously described under data collection and analysis) to construct an overview of co-creation and how it appeared in the context of the program evaluation (Table 1). The analysis also identified two additional elements for integration into the existing co-creation framework (Table 2).

## Co-creation experiences of researchers, TSO stakeholders and funders

The thematic analysis identified three broad themes, including (1) understanding the language and practice of co-creation, (2) perceptions of trust formation and (3) the value of co-creation opportunities. Each theme is presented below, utilising verbatim quotes.

1. Understanding the Language and Practice of Co-creation

Researchers, TSO stakeholders and funders perceived collaborative activities with different levels of understanding about co-creation and the activities within. With their continued involvement in implementing the framework, researchers were well acquainted with the concept of co-creation and its four collaborative activities at a theoretical level, with TSO stakeholders and funders less so. In contrast, TSO stakeholders understood “doing” co-creation as the co-activities tended to be part of their usual workday, even if they weren’t always able to label them the way the researchers had initially conceptualised. When asked about the language of co-creation, TSO stakeholders focused on the term co-design as what they were most familiar with;

*I worked with the [different suicide prevention activity] up in [a close regional town], and they all use it. Black Dog Institute [university-based suicide prevention institute] uses it, and health use it, and I’m straight out of Uni from last year, and it was all at Uni as well* (TSO stakeholder, 2020)

With co-creation, TSO stakeholders could identify instances during the co-design phase when the group began shaping tangible components of Eclipse. In one example, a participant observes the differing components of the US program compared to what was planned in Australia;

*I think co-design may…we may have reached that with Didi Hirsh because they had already come up with their design and their theories, but it was clinical. So, what I wanted was a non-clinical version of that, and I wanted Australian research that was able to support their research or not. So, I think that that’s how I see it.* (TSO stakeholder, CGD, 2020)

The approach taken when reviewing the US version of the program demonstrated a clear understanding of the need to adapt (and design) the program to meet the needs of end-users, as this TSO stakeholder expresses:

*Australia and America had two different environments. So, I think the American side at the time had a much lower appetite for risk than my particular centre, which had a higher appetite for risk, where DD Hirsch had a voluntary catch-up telephone call if they needed it. We made it mandatory, and from that, a social network was formed, and I think it was because the tyranny of distance we didn’t have. So, I think that our ability to adapt and change depends on the actual environment where whatever the research is about is* (TSO stakeholder, CGD, 2020)

During the CGD, participants in the discussion identified activities before the ‘co-ideation phase’. In this initial phase, a peer worker with lived experience (one of the TSO stakeholders) suggested creating a community program to meet the needs of people who have attempted suicide. This initial step was instrumental in the TSO making inquiries into establishing the Eclipse program. Here a TSO stakeholder described being unsure of whether similar programs existed.

…*So, I think from there, we went into the idea and exploration. We had no idea what was out there, but it was really not about doing something new. It was really about trying to find out what was out there *(TSO stakeholder, CGD, 2020).

### Perception of trust formation

Before the commencement of the project (in the newly identified pre-co-creation phase), researchers were consulted and invited to join the project. An existing working relationship between a funder and one of the researchers, in which trust already existed, prompted the invitation for collaboration. Complementary skills and expertise at the right time and a commitment to working collaboratively to achieve the stated objectives made for a successful outcome. The familiarity between researchers, TSO stakeholders, and funders enhanced the level of trust:

*Good fortune statement is really integral in research process because sometimes you do have to stumble across something in order to see the connection, and it’s not always deliberate, isn’t it?* (Researcher, CGD, 2020).

The success of the collaborative partnership and the program depended upon the development of trust between all involved. One of the researchers involved in the program confirmed how trust was integral to the relationship:

*It was like they all… the next person that everyone sought out, they knew that they were like them. So, like (manager) knew that (researcher) could be trusted, and then (manager) knew that you and I could be trusted and then when you and I work on stuff, then you know we can identify what’s going on. And then, with (TSO stakeholder) coming on board, it was really clear very early on that she was the right type of person, but that’s almost the big deficit with the other sites, is because they don’t have that drive or passion or curiosity to do this. They’re just doing it because it’s day to day business.* (Researcher, post-CGD reflective discussion, 2020)

The funder was also flexible in adjusting outcome expectations over time as external factors and participant engagement changed service delivery. A sense of trust between stakeholders was also related to the ability of the research team to respond to TSO stakeholders’ pressures:

*It is such a relief to have supportive researchers that our understanding of how the groups change and evolve, that the groups are impacted by droughts, fires, floods and COVID. That some participants withdraw and can’t be followed up for research. It really feels that the co-design is designed around the participants and not just the data collection* (TSO stakeholder, post CGD email, 2021).

The foundation of trust was also the basis for knowledge exchange between TSO stakeholders and researchers. In this instance, the TSO stakeholder reflects on how researchers used their knowledge of service delivery:

*I believe [TSO] helped the research understand nuances of all aspects of service delivery, from establishing the appropriate research paperwork to recruitment, appropriate training and support to the consumers of the service themselves and the barriers that might cause consumers anxiety. This helped the researchers design elements embedded in their research that navigated many of the barriers that might have come up* (TSO stakeholder, CGD, 2020).

### The value of co-creation opportunities

Through the growth of solid, trusting relationships, the co-creation process sparked several ‘spin-off’ activities, notably those initiated by people with lived experience.

For example, when asked if the TSO would continue to use co-creation, as reported by a TSO stakeholder:

*It [the process of co-creation] has inspired us to continue to grow our lived experience of suicide peer support workers. We have developed a Hospital to Recovery program based on peer support and lived experience*. (TSO stakeholder, CGD, 2020)

Another value-added opportunity generated through co-creation was how it provided TSO stakeholders with visions of hope for future services and programs:

*We would like to engage in more co-creation projects and programs to give reliability and validity to how we are delivering services. It has encouraged us to evolve Eclipse as many of them want to stay engaged after doing one or two cycles of Eclipse*. (TSO stakeholder, post CGD email, 2021)

In the program’s early stages, one of the major stumbling blocks in TSO stakeholders’ understanding of co-creation was how co-creation was viewed “through the lens [or context] of experience”. In this instance, from the perspective of TSO stakeholders, the main focus was developing the program alongside peer workers with lived experience and consumers. Co-creation activities like participant data collection (for the evaluation the researchers were conducting) received less attention. The data collection process was, in fact, a steep learning curve for researchers and the TSO stakeholders, where everyone involved held differing priorities. While TSO stakeholders were aware of the importance of evaluation, their main concern was service provision. As described by a TSO stakeholder:

*I felt that we got the curriculum here, but we really need…it’s about the participants. It’s about what they want, so we needed so we needed to be able to expand on that but stick to the curriculum but expand and have…it’s their group. It’s their group. We want to hear from them* (TSO stakeholder, CGD, 2020).

TSO stakeholders’ perception of data collection hinted at the “messiness” of the process and how, over time, their views changed through experience:

*[A] fear of mine as well, only because it’s the being thing, if you’re not in the frame of mind where you should be, tick, tick, tick [for the evaluation survey]…just to get rid of it. But then you’re sort of, well, if I don’t do this, we’re not going to learn what we need and basically, why did I set the group up in the first place. So, I had to change my judgement and my views on it as well to be able to sort of do it but just reading through a form* (TSO stakeholder, CGD, 2020).

The suggestion of messiness continued with trying to manage the data collection process and keeping participants engaged over time. However, solutions were also presented, in this case recommending additional administrative support:

*Following up participants one month and six months after groups, some disengage and no longer want to participate. Some participants were hard to engage online. Participants with attention issues – find it hard to participate with surveys in a group setting (too distracted). Time—it would be good to have more admin assistance* (TSO stakeholder, CGD, 2020).

With TSO facilitators focused on service delivery, it took some time for them to appreciate the purpose of integrating the data collection into the service delivery and the link between data fidelity, intervention effectiveness and quality improvement of the service;

*In order to achieve these ends, we needed to be fully aware of how and why of the research and evaluation process to ensure volunteer/participant buy-in and the data was collected in the correct way* (TSO stakeholder, post CGD email, 2021).

TSO stakeholder appreciation increased as they learned how research data represents an opportunity to create change. For instance, funders discuss the issues with a lack of complete data and the advantages of a larger sample size:

*So, the trends (in the data) are really helpful, but you know, the sort of…you can make a stronger case when you’ve got enough people, enough of a big sample, to be able to say okay, it’s significant. You know, so, that’s an extra level of strength in terms of sort of, you know, laying claim to this being a really effective program and therefore, you know, which we should be sort of top of the list when it comes to funding opportunities* (Funders, CGD, 2020).

And in the end, TSO stakeholder appreciation for co-creation and its benefits were described as follows;

*This research better captured the experience of those with lived experience were and what their aims for the program were. This helped us design the program and helped the researchers define the scope of the research* (TSO stakeholder, post CGD email, 2021).

## Discussion

Reflecting on the implementation and evaluation of the program and the researcher and TSO stakeholder perspectives captured in the collaborative discussion, it became clear that the co-creation framework represented a two-way open system with interactions between the internal and the external. With an open system, the framework, over time, was the subject of several conceptual changes as it adapted to the changing environment [25].

Table 1 operationalises the activities performed within the application of the co-creation framework to the case study, identifying barriers and challenges documented and described in evidence collected from pilot testing of the Eclipse program in 2017 through to the feedback collected in 2021.

**Table 1:** Researchers and TSO staff perspective—2017–2021

	Co-ideation	Co-design	Co-implementation	Co-evaluation
Stakeholder involved	TSO stakeholders Peer workers with LEX Researchers Funders	TSO stakeholders Peer workers with LEX Researchers Funders	TSO stakeholders Peer workers with LEX Researchers	TSO stakeholders Peer workers with LEX Researchers
Tools/process	Model adapted from USA support group	Data collection procedures Streamline communication using one email channel	Data collection flow charts Facilitator end-of-session prompts Process of sharing files Risk protocols Participant intake forms	Group survey checklist Attendance records Survey completion record
Opportunities	TSO stakeholders actively participated in the design and adaptation of the program	Researchers and TSO stakeholders share mutual knowledge and experience TSO stakeholders provided feedback on using measures in practice	TSO stakeholders developed awareness of how the program could be adapted over time whilst ensuring research integrity Consistency of service and feedback loop between TSO staff, peer workers and consumers	TSO stakeholders realised the value of the evaluation process and how results could result in benefits (increased funding) etc.
Barriers	Differences in researcher-stakeholder agendas can impede decision-making processes Delays in receiving ethics approval due to the sensitive nature of the research	Differences in the level of understanding between researchers and stakeholders about due process in adhering to research aims and rigour Understanding the validity of measurements	Awareness of the importance of data collection and its impact on program evaluation Reliance on researchers to manage data, changing of program components without joint discussion COVID pandemic	The lack of participant data prevents TSO stakeholders from demonstrating positive program outcomes COVID pandemic
Lessons learnt	Appreciation of individual stakeholder expertise, skills and role demands and what they bring to co-creation	Consistent and open communication is required, especially regarding decisions impacting the research process	Implement administrative resources to act as an intermediary between the researcher and TSO Stakeholders’ participation in the data collection to cement relationships between stakeholders	Participation of stakeholders in the evaluation process can help generate new innovative opportunities

Applying the framework to the case study highlighted two new areas not previously identified. These changes include the addition of a (1) pre-co-creation stage, the possibility of (2) spin-off opportunities and the (3) reiterative processes across the research cycle. First, in the original framework, there was no emphasis on the entry point to co-creation. In this case study, the framework was not considered a linear process with a fixed starting point beginning with “co-ideation” and ending with “co-evaluation”. Co-creation originated external to the co-creation process and depended on the agreement between collaborators to work together to achieve an identified common goal. Second, the co-creation process generates spin-off opportunities which are then feedback in the co-creation process. Additional sites, as well as unexpected events, required prompt flexibility. During COVID-19, along with several natural disasters (multiple floods and fires that resulted in widespread evacuations and dislocation) occurring during the collection period, there was a need to move to online delivery.

As a consequence of co-creation, these spin-off opportunities drew on the knowledge of existing stakeholders, enabling a more efficient and effective means of working together on these new projects. Third, as the stakeholders and researchers carried out the simultaneous implementation and evaluation of the program, the researcher responded and modified the design in real-time to accommodate changing needs. In particular, training guides for TSO stakeholders on data collection and discussions on improving the readability of surveys used in the data collection process and replacing paper forms with online data collection such as web-based surveys.

Currently, there is minimal evidence of TSOs adopting co-creation as a translation model [17]. Operationalising the co-creation framework to an activity provides insight into how this form of collaboration occurs and allows for assessing whether this method reduces the research-practice gap. While this activity identified issues associated with implementing some co-activities (co-ideation, co-design, co-implementation and co-evaluation) within the context of a program and practice setting, we identified some barriers and opportunities for applying co-creation to a health intervention. Overall, our findings highlighted three main points: (1) the messiness of co-creation, (2) the evolution of the co-creation framework, and (3) how trust served as a driving force of good fortune and good governance.

First, the study’s findings speak to the complexity of co-creation where, to the uninitiated, it appears as a messy concept to implement and practice. The “messiness” of co-creation occurs on several levels, namely within the process of “doing” co-creation, where participants (subtle and intangible process) and the relationship between researchers and TSO stakeholders. There is messiness in the process when co-activities overlap, with no clear line separating each activity from the next. Across the four phases of the research cycle, researchers and TSO stakeholders engaged in multiple rounds of creating ideas and designing solutions. Co-creation’s iterative design is in direct contrast to the linear and systematic process commonly associated with traditional research [26]. While the straightforwardness of a traditional research approach has its appeal in being researcher-led and systematic, co-creation has the advantage of its reiterative processes of co-creation, which work to resolve any methodological problems. In describing the implementation and evaluation of the program, the TSO stakeholders expressed this ‘messiness’ of the process and the management of stakeholder relationships.

For those TSO stakeholders participating in the collaborative discussion, they perceived the process of program implementation and evaluation “*through the lens of experience*”. As the project evolved, we learned and became acutely aware of the differing priorities, an issue highlighted in the data collection process. Stakeholder relationships also encountered complexity in managing power and equity amongst researchers and TSO stakeholders. In line with previous studies [27], the involvement and participation of researchers and TSO stakeholders across the four co-creation research cycles varied at different times and for different tasks. As evidenced in this study, the greater the level of investment in the program by TSO stakeholders, the higher the rate of participation.

Moreover, stakeholders who invested more in co-creation had increased knowledge and expertise about program evaluation, which helped drive innovation. This finding extends the work of previous stakeholder research, claiming that stakeholder commitment to program evaluation positively impacted the utilisation of evaluation findings [27] and consolidated their understanding of their roles in practising co-creation [28]. The quality of the collaborative partnership was dependent on the building of mutual trust [28]. Trust formation encouraged flexibility and adaptability to change for those involved in the program. However, it could be theorised that the “messiness” of co-creation may, over time, dissipate as researchers and TSO stakeholders become more proficient at implementing the co-creation framework and/or applying it to other contexts settings. Identification of these challenges has provided a solid foundation for re-assessing the proposed co-creation framework and extending it by including critical activities of each stage, namely roles members may undertake and, importantly, measurable outcomes from each stage so that continuous monitoring of the progress can be undertaken. As there is no temporal limitation, nor does the model require a linear progression, measurable outcomes from each stage may assist future application of this model to assess where further work is required in these collaborative activities. Given the findings from this case study reported on the application of the co-creation framework, a revised, updated framework is presented in Table 2, which includes the additional elements and critical tasks associated with each component. Furthermore, this framework is presented in a format that can be used for other interventions for further testing and refinement.

**Table 2:** Co-creation of new knowledge in practice

	Key activities (initial discussions)	Identification of key players	Measurable outputs
Pre-co-creation Phase	Researchers and service providers connect through pre-existing relationships or organisations contacting universities to discuss the need for program evaluation. Internet searches for pre-existing programs are conducted by organisations or researchers and/or review statistics or records, service priorities or clinical guidelines	Researchers Service providers Service users Funders Policymakers (optional)	Relevant contractual documents signed by all parties. Contract research agreement Memorandum of understanding
Co-ideation	Discussion on the purpose of the project/intervention, including identification of barriers and solutions of collaboration, planning of the evaluation and applying the co-creation framework, includes agreeing on an approach and identifying and agreeing on roles – both of these may involve ongoing discussions between co-ideation and co-design	Service providers Service users Researchers Funders (optional) Policymakers (optional)	Agreement to work on common goals; evidence of this might include terms of reference, principles of working
Co-design	Reiterative process to assess what works and what doesn’t work Sessions to understand different roles/responsibilities and how change can be integrated into the routine delivery of services Stakeholders and researchers jointly collaborate on the research design planning and input into HREC (Human Resource Ethics Committee) application. Fluid ideas and creative thinking are required. Technical details (prototype) on data collection are documented but open to further revision. Provide a detailed description of the new service or program.	Service providers Service users Researchers Funders (optional) Policymakers (optional)	Protocol for program evaluation completed and registered HREC application completed Technical details about the evaluation documented, including clear and tangible goals and criteria for success
Co-implementation	Implement the co-designed program as per the protocol Before the collection of data need to make clear how researchers and stakeholders can be influential in the partnership, so the validity and reliability of the data are maintained and balanced with the demands of the organisation delivering services	Service providers Service users Researchers	Regular meetings for ongoing review of the process Evidence of tangible goals being achieved
Co-evaluation	Embedding of the data collection process into the delivery of services. The data collection process may require adjustments and discussion on streamlining the process if unexpected barriers (including both internal and external barriers) Measure the process of data collection against KPIs.	Service providers Service users Researchers Funders (optional) Policymakers (optional)	Data was collected by using evidence-based questionnaires and scales during the delivery of programs and services or treatments Researchers provide evidence in a manner that is usable and practical for the organisation

NB: Operational definition adapted from Pearce et al. [10]

### Good fortune and good governance

The findings raise the critical question of whether good fortune or good governance led to the successful implementation of co-creation in this example. This paper argues that it is both. Good fortune suggests the outcome was generated by luck, with stakeholders being “in the right place at the right time”. However, given that the researchers and stakeholders shared a pre-co-creation relationship, the primary driver of good fortune was the social capital created outside the co-creation framework. These connections appeared to generate favourable conditions for speeding up the process of forming collaborative partnerships between researchers, TSO stakeholders and peer workers with lived experience.

While social capital plays a key role, various mediating factors in this study appeared to contribute to increased levels of trust and reciprocity. These included (1) the complementary expertise and skills of each researcher and stakeholder, (2) the level of commitment by researchers and stakeholders to continue the evaluation despite facing critical events (COVID-19 pandemic, changes in key staff, loss of funding), (3) regular contact through frequent meetings and correspondence and, (4) the sharing of explicit and tacit knowledge resulting in greater collaborative reciprocity. These findings are consistent with those identified in research on trust-based social capital, where such factors lead to higher levels of trust and innovative practice [29]. Although some claim that trust-based collaboration can be developed from the ground up [30], other evidence suggests that trust takes longer to crystallise when social capital is reduced [29]. A lack of social connection between stakeholders can also lead to increased conflict, disrupting the implementation process [29]. Besides trust and social connection, co-creation is successful when good management and research governance are practised. Studies on the dynamics of research collaboration [31] suggest that good management involves committing to achieving project goals and outcomes and ensuring stakeholders are involved in decision-making processes [32]. As evidenced in the transcripts of this case study, the presence of natural disasters and pandemics created challenges to collecting data and meeting project deadlines. Regardless, the flexible nature of researchers and stakeholders and frequent communication allowed for achieving goals. Studies on good management also perceive regular communication between researchers and stakeholders as important as collaborative relationships. While the researchers and TSO stakeholders were not necessarily sharing the same physical space, collaboration occurred virtually through online meetings or by phone and email. Although, the disadvantage of the distance between collaborators makes it challenging to engage in casual conversations that generate new ideas, good management and attention to the virtual space inhabited by researchers and TSO stakeholders have enabled relationships to evolve.

### Strengths and limitations

The methodological rigour built into the case study is a strength of the paper. Over the four year course of the project, researchers, funders and TSO stakeholders, including peer workers with lived experience, regularly discussed, through telephone conversations and formal meetings, ongoing issues relating to the implementation and evaluation of co-creation of the Eclipse program. The transmission of information between researchers and stakeholders worked to triangulate or corroborate the findings, a process known as member/peer or peer checking [33]. In qualitative research, member/peer checking enhances validity and trustworthiness in the case study process by reducing the possibility of researcher bias and improving the validity of the case study process [33]. Also, verifying results and detecting bias were made more accessible by triangulating evidence from multiple sources used in this case study [34]. Researchers should consider the generalisability of the findings to other suicide prevention programs with caution. The specificity of the case and the participant sample size may limit its applicability to other TSOs. However, TSOs delivering health interventions are encouraged to implement co-creation to improve generalizability.

### Implications for professional practice, policy and research

For TSO practitioners delivering mental health and suicide prevention services and policymakers, co-creation offers several benefits. Adopting a co-creation framework satisfies a global “whole of government” initiative where the government sees the benefit of forming multisector collaborations between researchers, TSOs and peer workers with lived experience [35]. By collaborating, researchers may be able to reduce the gap between knowledge creation and its implementation into practice.

Unlike many other translation models, a central principle of the co-creation framework is embedding the process of data collection into the routine delivery of services. TSO stakeholders may improve the quality of services by simultaneously implementing the new knowledge as it’s developed. Evidence suggests that integrating data collection and service delivery may lead to higher stakeholder participation in the research process and increased investment by TSO stakeholders in designing and delivering those programs [36]. As indicated in this study, co-creation allows for flexibility and creativity in its design by readily adapting to the changing needs of the TSO environment [10]. For policymakers, one key benefit of co-creation is the potential for an increase in the number of TSO evaluations producing high-quality evidence. Governments rely on the production of knowledge to support informed decisions and policy planning around health services and interventions [17]. An increase in evaluations may produce a higher rate of relevant and timely evidence for implementation into policy. Finally, for researchers, this study contributes more than a theoretical approach or an empirical observation to the advancement of knowledge in co-creation and collaborative practice. By applying the framework to a TSO delivering suicide prevention services, we have offered a pragmatic, step-by-step approach to implementing the framework and identifying improvement areas. For instance, ensuring TSO practitioners share a common language and meaning with researchers around the definition of core concepts such as co-ideation and co-creation. While terms such as co-ideation were not necessarily recognisable by stakeholders at a theoretical level, evidence from the shared discussion between researchers and stakeholders indicates, at a practical level, some semblance of understanding by stakeholders of popular concepts such as co-design. Misunderstandings around the definition of the co-creation framework can impede the capacity of researchers and stakeholders to engage in an informed critical discussion on co-creation and its four collaborative processes. Building awareness amongst mental health and suicide prevention stakeholders is necessary to implement the co-creation framework successfully.

## Conclusion

As demonstrated in this case study, co-creation is a viable framework for creating new knowledge, increasing research uptake into practice and improving outcomes of health interventions in suicide prevention. There was little evidence, to date, of co-creation’s effectiveness as a new and untested method. This approach—and this example of a four-year project—does not sit easily with current government funding strategies, which promote short-term funding cycles and the production of rapid results. While methodologically, Randomised Controlled Trials (RCTs) have become the accepted approach to achieving a gold standard, as alluded to in this case study, it is possible to conduct robust research whilst remaining human and pragmatic. Funding structures should consider co-creation’s long-term benefits, particularly in sectors where evaluations are less likely to be conducted, such as TSOs. Further research is required to test the co-creation framework in similar environments to expand our understanding of its impact on stakeholders and effectiveness in improving service users’ outcomes.

## Data Availability

The datasets used and/or analysed during the current study are available from the corresponding author upon reasonable request. All data and materials supporting the findings reported in the paper are located at the University of New England. The audio-recorded consultations are not possible to share because the individual privacy of participants would be compromised.

## Abbreviations

CGD: Collaborative group discussion
HREC: Human research ethics committee
LEX: Lived experience
RCTs: Randomised controlled trials
PPI: Public and participant involvement
NSW: New South Wales
TSOs: Third-sector organisations

## References

[1] Straus SE, Tetroe J, Graham I (2009). Defining knowledge translation. CMAJ Can Med Assoc J.

[2] Gonzalez B, Miguel A (2004). Bridging the implementation gap in health systems research: commentary. Bull World Health Organ.

[3] Milat AJ, Bauman A, Redman S (2015). Narrative review of models and success factors for scaling up public health interventions. Implement Sci.

[4] Nowotny H, Scott PB, Gibbons MT (2013). Re-thinking science: Knowledge and the public in an age of uncertainty.

[5] Lewig K, Arney F, Scott D (2006). Closing the research-policy and research-practice gaps: ideas for child and family services. Family Matter.

[6] Sanson A, Stanley F. Improving the wellbeing of Australian children and youth: The importance of bridging the know-do gap. Bridging Know Do Gap. 2010;3.

[7] Bach-Mortensen AM, Montgomery P (2018). What are the barriers and facilitators for third-sector organisations (non-profits) to evaluate their services? A systematic review. Syst Rev.

[8] Robinson T, Bailey C, Morris H, Burns P, Melder A, Croft C (2020). Bridging the research–practice gap in healthcare: a rapid review of research translation centres in England and Australia. Health Syst Policy Res.

[9] Horsfall J, Cleary M, Hunt GE (2011). Developing partnerships in mental health to bridge the research–practitioner gap. Perspect Psychiatr Care.

[10] Pearce T, Maple M, Shakeshaft A, Wayland S, McKay K (2020). What is the co-creation of new knowledge? A content analysis and proposed definition for health interventions. Int J Environ Res Public Health.

[11] Werker E, Ahmed FZ (2008). What do nongovernmental organisations do?. J Eco Perspect.

[12] Kareithi R, Lund C (2012). Review of NGO performance research published in academic journals between 1996 and 2008. S Afr J Sci.

[13] Fine AH, Thayer CE, Coghlan A (2000). Program evaluation practice in the non-profit sector. Non-profit Manag Leadersh.

[14] Australian Government Productivity Commission. Productivity commission, mental health, inquiry report. Canberra: Australian Government; 2020. Report No.: 95 Available from: https://www.pc.gov.au/inquiries/completed/mental-health/report.

[15] Greenhalgh T, Jackson C, Shaw S, Janamian T (2016). Achieving research impact through co-creation in community‐based health services: literature review and case study. Milbank Q.

[16] Kozak A (2019). The effectiveness of the public services co-production process–results of a systematic literature review. Econ Entrep Manag.

[17] Pearce T, Maple M, Wayland S, McKay K, Woodward A, Brooks A (2022). A mixed-methods systematic review of suicide prevention interventions involving multisectoral collaborations. Health Res Policy Sys.

[18] Lifeline Mid Coast. Lifeline mid coast 2022. Available from: https://lifelinemidcoast.org.au/.

[19] Maple M, Wayland S, Pearce T, Bhullar N (2020). Protocol for the evaluation of a non-clinical, community based facilitated support group for people who have previously attempted suicide. ResearchGate.

[20] Didi Hirsch Mental Health Services. Manual for support groups for suicide attempt survivors Los Angeles, CA.: Didi Hirsch Mental Health Services; 2014. Available from: https://zerosuicide.edc.org/resources/resource-database/manual-support-groups-suicide-attempt-survivors.

[21] Merriam S. B. Case study research in education: a qualitative approach. Jossey-Bass; 1988.

[22] Crowe S, Cresswell K, Robertson A, Huby G, Avery A, Sheikh A (2011). The case study approach. BMC Med Res Methodol.

[23] Kiger ME, Varpio L (2020). Thematic analysis of qualitative data: AMEE guide no. 131. Med Teach.

[24] Braun V, Clarke V (2006). Using thematic analysis in psychology. Qual Res Psychol.

[25] Hieronymi A (2013). Understanding systems science: a visual and integrative approach. Syst Res Behav Sci.

[26] Roberts L (2012). Community-based participatory research for improved mental healthcare: a manual for clinicians and researchers.

[27] Snijder M, Shakeshaft A, Wagemakers A, Stephens A, Calabria B (2015). A systematic review of studies evaluating Australian indigenous community development projects: the extent of community participation, their methodological quality and their outcomes. BMC Public Health.

[28] Dong B, Evans KR, Zou S (2008). The effects of customer participation in co-created service recovery. J Acad Mark Sci.

[29] Filieri R, McNally RC, O’Dwyer M, O’Malley L (2014). Structural social capital evolution and knowledge transfer: evidence from an Irish pharmaceutical network. Ind Mark Manag.

[30] McKnight DH, Cummings LL, Chervany NL (1998). Initial trust formation in new organizational relationships. Acad Manag Rev.

[31] Bozeman B, Gaughan M, Youtie J, Slade C, Rimes H (2015). Research collaboration experiences, good and bad: dispatches from the front lines. Sci Public Policy.

[32] Oliver K, Kothari A, Mays N (2019). The dark side of co-production: Do the costs outweigh the benefits for health research?. Health Res policy Syst.

[33] Birt L, Scott S, Cavers D, Campbell C, Walter F (2016). Member checking: A tool to enhance trustworthiness or merely a nod to validation?. Qual Health Res.

[34] Yin RK (2012). Applications of case study research California.

[35] Pearce T, Maple M, Wayland S, McKay K, Woodward A, Shakeshaft A (2022). Evidence of co-creation practices in suicide prevention in government policy: a directed and summative content analysis. BMC Public Health.

[36] Okul E, Nyonje R (2020). Examining stakeholder involvement in the evaluation process for program improvement. Int J Res Bus Soc Sci.

